# Evaluating Serum Copeptin as a Promising Biomarker for Predicting Acute Ischaemic Stroke Severity: A Hospital-Based Study on Strokes

**DOI:** 10.7759/cureus.63700

**Published:** 2024-07-02

**Authors:** Feyisayo Alabi, Ikechukwu Chukwuocha, Ernest Nwazor, Victor Onyenokwe

**Affiliations:** 1 Internal Medicine, University of Port Harcourt Teaching Hospital, Port Harcourt, NGA; 2 Neurology, University College Hospital, Ibadan, NGA; 3 Neurology, Rivers State University Teaching Hospital, Rivers State, NGA; 4 Cardiology, University College Hospital, Ibadan, NGA

**Keywords:** blood-based biomarker, infarct size, nihss (national institutes of health stroke scale), acute ischaemic stroke, copeptin

## Abstract

Background: Stroke is the second cause of mortality and the foremost leading cause of disability globally. Many potential biomarkers have been described to contribute to prognosticating the severity in the acute phase of stroke as well as help with risk stratification. Copeptin, an inactive peptide that is produced in an equimolar ratio to arginine vasopressin and adequately mirrors an individual’s stress response to acute illnesses like acute ischaemic stroke as evidenced by elevated or increasing levels is being explored in this study to determine its relationship with acute stroke severity and infarct size on admission.

Methods: This is a cross-sectional study of 80 neuroimaging-confirmed acute ischaemic patients who presented within seven days of symptom onset and 80 control subjects. The ischaemic stroke cases had stroke severity and infarct volume determined on admission by the National Institute of Health Stroke Scale (NIHSS) and neuroimaging (brain CT/MRI). A baseline serum copeptin level was measured in the study subjects. Spearman correlation and Kruskal Wallis test were used to determine the relationship between serum copeptin level with admission NIHSS and infarct size respectively. The receiver operating characteristic (ROC) curve was calculated to determine the sensitivity and specificity of copeptin to predict severity and outcome.

Results: The mean age of the study group was 61.3 ± 12.7 years with 55.0% males and 45.0% females. The serum level of copeptin was significantly higher in the stroke cases with a median of 28.6 pmol/L (interquartile range (IQR)- 15.4-31.6 pmol/L) versus 8.8 pmol/L (IQR- 3.2- 10.7 pmol/L) among the stroke-free controls (p= 0.001) at a statistically significant level. There was a weak correlation between copeptin and NIHSS calculated at admission to measure stroke severity (r- 0.02, p= 0.873). Patients with infarct sizes in the fourth quartile (infarct sizes greater than 18.78 cm^3^) had higher copeptin levels, though this was not statistically significant (H= 2.88; p= 0.410). Admission serum copeptin did not show a statistically significant prognostic value in predicting stroke severity and mortality in stroke patients who presented within seven days of symptom onset with an area under curve (AUC) of 0.51 (95% CI: 0.36-0.65; p= 0.982).

Conclusion: In this study, copeptin was higher among the stroke cases compared with the stroke-free controls which suggests a significant prognostic value in risk stratification in the acute phase of stroke; however, this did not significantly correlate with stroke severity and thus warrants further study in this field to elucidate it’s fascinating potential as a prognostic biomarker (especially in the acute period) as this may enable allocation of a better-focused therapy for stroke patients.

## Introduction

Stroke is one of the leading causes of death in Nigeria as well as globally and represents the foremost cause of disability among the adult population [1]. Stroke burden will continue to rise globally, particularly in low-income countries, when one considers the age profile of the majority of stroke patients as well as the world's continuously growing population and dramatically increasing life expectancy [2]. The burden of stroke in Africa is huge as evidenced by records of having the highest incidence, prevalence and case fatality of stroke. Presently, the prevalence of stroke in Nigeria is between 1.14-7.7 per 1000. From 2004- 2021, hospital-based research from various parts of Nigeria revealed a 30-day case fatality rate of about 21.2-40% [3-6] For optimal care and efficient use of the healthcare resources at hand, early evaluation of the severity of the disease, the risk of complications, and the prognosis is crucial [7].

Several neurophysiological methods and clinical assessment scales have been developed and shown to be predictive in determining the severity of stroke [8,9]. The National Institutes of Health Stroke Scale (NIHSS) is one of the recognized clinical scores with prognostic potential [10]. A biomarker is defined as a characteristic that is objectively measured and evaluated as an indicator of normal biological processes, pathogenic processes, or pharmacologic responses and is accepted as a valuable tool for diagnosis, prognosis, and possible therapeutic targets in different disease entities [11,12]. In the context of an acute ischaemic stroke, the assessment of selected blood biomarkers may be useful in making a diagnosis, predicting severity, and guiding management and outcome post-stroke. Meanwhile, clinical data alone has not shown to be adequate for precise prognostication after acute ischaemic stroke due to the variability of stroke clinical manifestation. Copeptin, a prognostic biomarker that is more stable and co-released with arginine vasopressin, which additionally mirrors an individual’s stress response to acute illnesses like acute ischaemic stroke as evidenced by elevated or increasing levels in the setting of acute ischaemic stroke may serve as an adjunctive tool in this setting as a biomarker-based approach to help predict the severity of acute ischemic stroke with a good degree of reliability [13-15]. This novel biomarker is a 39 amino acid glycopeptide. It is synthesized mainly in the paraventricular neurons of the hypothalamus and in the supraoptic nucleus as a C terminal part of the precursor protein. Pre-provasopressin after cleavage has been found through studies to predict severity in ischaemic stroke and, in addition, improves the diagnostic accuracy of standardized stroke severity and outcome clinical scores like the National Institutes of Health Stroke Scale (NIHSS) [16,17]. When plasma copeptin is tested early in acute stroke, it provides a more accurate prognostic evaluation of acute ischemic stroke severity, according to a systematic review and meta-analysis of several observational studies evaluating the predictive function of copeptin in stroke patients [18]. Additionally, elevated copeptin concentration following acute ischaemic stroke has been found to be associated with larger infarct size and portends poorer prognosis in these individuals. This study was aimed at exploring the relationship between serum copeptin levels on admission with ischaemic stroke severity and infarct volume.

## Materials and methods

In this cross-sectional study, 80 consecutive patients with acute ischemic stroke and 80 age and sex-matched controls who met the inclusion criteria (age >18, first ischemic stroke, and admission to the hospital within one week of onset of stroke) were included. Stroke was defined according to the World Health Organization as “rapidly developing clinical signs of focal/global disturbance of cerebral function, with symptoms lasting 24 hours or longer with no apparent cause other than of vascular origin”. Cerebral infarction was confirmed with neuroimaging (CT or brain MRI). All subtypes of ischemic stroke were included. This study was approved by the University of Port Harcourt Teaching Hospital Institutional Ethical Committee with approval number UPTH/ADM/90/S.11/VOL.XI/1109.

The cases included in this study were subjected to the following: clinical evaluation, careful history taking, general medical examination including vital signs and cardiac assessment, and thorough neurovascular examinations. Based on their clinical presentation and the findings of diagnostic studies, the subjects were categorized using The TOAST classification (Trial of Org 10172 in Acute Stroke Treatment), which distinguishes five subtypes of ischemic stroke: large-artery atherosclerosis, cardioembolism, small-vessel occlusion, a stroke of other determined aetiology, and stroke of unknown aetiology. The National Institutes of Health Stroke Scale (NIHSS) was used to assess the severity of stroke at presentation which was categorized as mild (score: 1-4), moderate (score: 5-15), moderately severe (score: 16-20) and severe (21-42).

Laboratory evaluation: Blood glucose, electrolyte, renal function, complete blood count, and lipid profile assessments were performed on patients and control groups as standard procedures. At the time of presentation, blood samples for determination of serum copeptin were drawn into tubes containing potassium ethylenediaminetetraacetate (EDTA), kept for 5 mins, and then centrifuged. The separated serum was stored at or below -80 °C. Serum copeptin level was subsequently determined in both cases and the control group using enzyme-linked immunosorbent assays (ELISA) and a Stat Fax 2100 Microplate Reader (Elabscience, Houston, Texas).

The radiological assessment consisted of an MRI stroke protocol and brain computed tomography (CT) to determine the location and volume of the infarction. The formula A × B × C/2 was utilized to determine the infarct volume. B represents the measured perpendicular diameter of the infarction, C is the total thickness of the slices where the infarction was observed, and A is the largest diameter.

Data was analysed using the Statistical Package for Social Sciences (SPSS) version 24.0 (IBM Corp., Armonk, USA). Continuous variables were presented as mean ± SD, median, and interquartile range when the data was not normally distributed. Categorical variables were expressed as frequencies and proportions. Comparison between groups was done using the Mann-Whitney U test and other relevant statistical tests were appropriate. Pearson correlation was used to test for possible correlations between quantitative variables. A p-value less than or equal to 0.05 was considered significant. Receiver operating characteristic (ROC) curve analysis was used to assess the prognostic role of copeptin level in relation to acute stroke severity.

## Results

A total of 160 participants completed the study, of which 88 participants were males (55.5%) and 72 participants (45.0%) were females (Table 1). Among the stroke patients, 47 participants were men (58.8%) and 33 (41.2%) were women, while 41 (51.3%) and 39 participants (48.7%) were men and women in the control group. The mean age of participants in the total study population was 61.312.7 years. The sociodemographic variables of the study participants are presented in Table 1.

**Table 1:** Sociodemographic characteristics of study participants

Characteristics		Study Groups		P Value
	Total N = 160 (%)	Stroke Patients N = 80 (%)	Controls N = 80 (%)	
Sex				
Male	88 (55.0)	47 (58.8)	41 (51.3)	0.340
Female	72 (45.0)	33 (41.2)	39 (48.7)	
Age				
< 40 years	6 (3.8)	3 (3.7)	3 (3.7)	0.661
40 - 49 years	21 (13.1)	8 (10.0)	13 (16.3)	
50 - 59 years	40 (25.0)	19 (23.8)	21 (26.3)	
60 - 69 years	56 (35.0)	28 (35.0)	28 (35.0)	
70 - 79 years	25 (15.6)	16 (20.0)	9 (11.2)	
> 80 years	12 (7.5)	6 (7.5)	6 (7.5)	
Age in years – Mean ± SD	61.3 ± 12.7	62.5 ± 12.3	60.1 ± 12.5	0.221

With respect to the comorbid conditions among the study participants, only hypertension and diabetes mellitus showed a statistically significant difference between the cases and the control group (p=0.001; p=0.002) respectively. The co-morbid medical conditions are presented in Table 2.

**Table 2:** Comorbidities among study participants

Characteristics		Study Groups		P Value
	Total N = 160 (%)	Cases N = 80 (%)	Controls N = 80 (%)	
Co-morbid medical conditions				
Hypertension				
Yes	128 (80.0)	75 (93.8)	53 (66.3)	0.001*
No	32 (20.0)	5 (6.3)	27 (33.8)	
Diabetes				
Yes	52 (32.5)	35 (43.8)	17 (21.3)	0.002*
No	108 (67.5)	45 (56.3)	63 (78.8)	
Heart Disease				
Yes	32 (20.0)	15 (18.8)	17 (21.3)	0.693
No	128 (80.0)	65 (81.3)	63 (78.8)	

**Figure 1:**
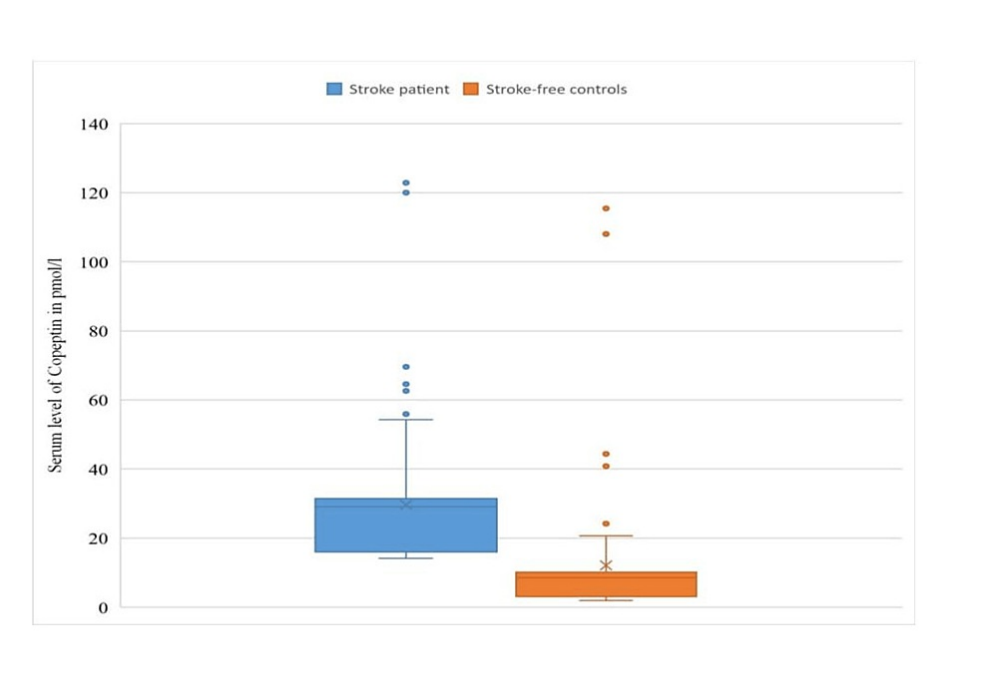
Box and whisker plot comparing the median serum copeptin level among stroke patients and controls

The median serum copeptin among stroke patients in this study was 28.6 pmol/l (15.4 to 31.6 pmol/l) and this was significantly higher (U-test = 812.0; p=0.001) among the stroke patients (Median = 28.6; IQR: 15.4-31.6 pmol/l) than the control participants (Median = 8.8; IQR: 3.2-10.7 pmol/l). However, further analysis did not reveal a significant association between serum copeptin and day post ictus in which the individual stroke cases presented with the first week of stroke (Kruskal Wallis ꭓ2= 11.44; p = 0.076). The comparison of serum copeptin levels between cases and controls is depicted in Figure 1 and Table 3.

**Table 3:** Comparison of serum copeptin among stroke patients and controls

Characteristics		Study Groups		Mann U test
	Total Median (IQR)	Stroke patients Median (IQR)	Controls Median (IQR)	
Serum Copeptin level in pmol/l	15.4 (8.4 – 29.8)	28.6 (15.4 – 31.6)	8.8 (3.2 – 10.7)	812.0 (0.001*)

Based on the TOAST categorization of stroke type, five patients had a cardioembolic stroke (6.3%) and had a median copeptin level of 28.4 pmol/l (IQR: 21.1-32.2pmol/l), 32 patients (40.0%) had small vessel disease with median copeptin level of 29.9 pmol/l (IQR: 16.1-30.9 pmol/l). Patients with large artery atherosclerosis (17.4%) and those with a stroke of undetermined aetiology (36.3%) due to incomplete evaluation had median copeptin levels of 27.8 pmol/l (IQR: 19.4-38.4 pmol/l) and 27.4 (IQR: 15.4-32.4 pmol/l), respectively (Table 4). The study did not find a significant statistical difference between the serum copeptin and the categories of the TOAST classification (Kruskal Wallis ꭓ2= 0.18; p - 0.981).

**Table 4:** Serum copeptin level among stroke patients at different levels of stroke severity IQR: Interquartile

Outcome	Frequency N = 80 (%)	Median (IQR)	p Value
Glasgow coma scale			
Mild (Score: 13 – 15)	54 (67.5)	28.6 (15.5 – 31.7)	0.962
Moderate (Score: 8 – 12)	19 (23.8)	22.08 (15.4 -32.6)	
Severe (Score: < 8)	7 (8.7)	30.0 (15.4 – 31.4)	
Stroke Severity by NIHSS			
Minor (Score: 1 – 4)	12 (15.0)	29.0 (15.2 – 31.3)	0.843
Moderate (Score: 5 – 15)	32 (40.0)	29.6 (15.4 – 32.3)	
Moderately severe (Score: 16 – 20)	11 (13.8)	25.3 (20.9 – 28.1)	
Severe (Score: 21 – 42)	25 (31.3)	29.5 (15.7 – 32.0)	

The study did not reveal any statistically significant relationship between serum copeptin levels among stroke patients with the different categories of stroke severity using the NIHSS rating scale (Kruskal Wallis ꭓ2= 0.83; p = 0.843). Furthermore, the ability of Serum Copeptin level to predict stroke severity with respect to NIHSS was very low as determined by the ROC. The area under the curve (AUC) was 0.51 for NIHSS (95%CI: 0.36 - 0.65; p = 0.982). 

**Figure 2:**
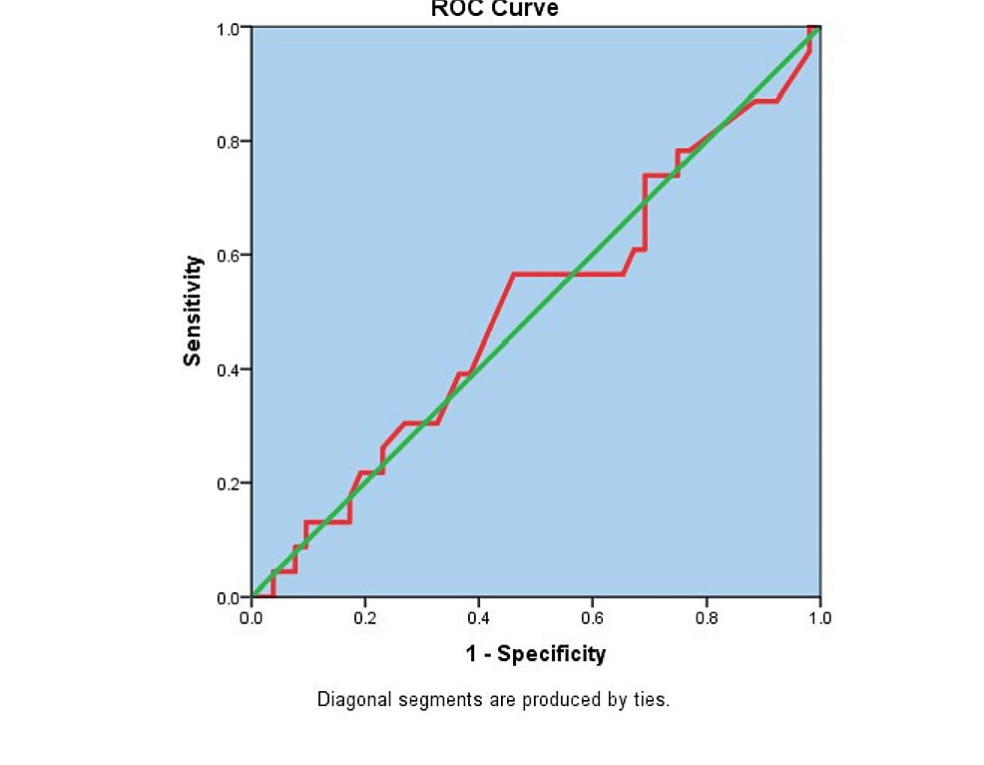
ROC curve for serum copeptin while predicting severity of stroke with respect to NIHSS among stroke patients ROC: Receiver operator characteristic; NIHSS: National Institute of Health Stroke Scale

Copeptin prognostic ability in relation to severity as categorized by NIHSS is as shown in Figure 2. The level of serum copeptin which showed the best prognostic value for predicting severity with respect to the severity grading by NIHSS was 29.04 mmol/L with only a sensitivity of 57% and specificity of 54% as shown in the Table 4.

Smaller infarct volumes predominated in the distribution of infarcts. Fifty percent of the stroke patients had infarcts that were less than 8.97 cm^3^ in size. The summary statistics for infarct size for each of the four quartiles and association with serum copeptin using the Kruskal Wallis test are shown in Table below. Patients with infarct sizes in the fourth quartile (infarct sizes greater than 18.78cm^3^) had higher copeptin levels. The patients with infarct sizes in the four quartiles did not differ significantly from one another statistically (H = 2.88; p-value = 0.410) as depicted in Table 5.

**Table 5:** Serum copeptin level and infarct size

Infarct size category (cm^3^)	n (%)	Mean	Standard Deviation	Median	IQR
Infarct size between 0 - 3.53 cm^3^	20	30.46	25.38	25.80	16.44
Infarct size between 3.54 - 8.97 cm^3^	20	30.64	13.79	29.52	6.42
Infarct size between 8.98 - 18.77 cm^3^	20	27.61	23.38	21.48	14.82
Infarct 18.78 cm^3^	20	80.59	165.96	28.96	9.64

## Discussion

The mean age of the study participants was 61.3 ± 12.7 years, and the number of stroke cases was highest in the seventh decade of life with the prevalence reducing thereafter. This is comparable to findings from several other studies in the region [19,20]. This study observed that more males than females were found in the study group. This was similar to the finding by Ekeh et al. in a study conducted in Jos, Nigeria, among acute ischaemic stroke patients and may be explained by the fact that most modifiable risk factors are hypertension, smoking, alcohol, and illicit drug use which are more prevalent in men [21].

In this study, serum copeptin levels were found to be higher in the stroke patients than in the controls, and this difference was found to be statistically significant. This was similar to the findings in studies by Dong et al., [22] Oraby and colleagues [23], and Kevin et al. [24] who reported serum copeptin levels of 15.8 pmol/L (IQR- 10.1-25.8) versus 4.3 pmol/L and 120.52 ± 45.7 pg/ml versus 76.51 ± 32.8 pg/ml in their stroke and control subjects respectively. In our study, the median (IQR) serum copeptin levels as found in the stroke cases vs the control population were 28.6 pmol/L (15.4-31.6 pmol/L) vs 8.8 pmol/L (3.2-10.7). This is not unusual as serum copeptin levels are found to be significantly higher in patients with acute illnesses such as stroke [25], and this is in agreement with several studies that observed approximate numerical rise in serum copeptin levels in acute stroke patients. [23,26,27]. This finding can be explained by the fact that copeptin, which is a stable peptide and co-secreted with arginine vasopressin (AVP) is a neuronal hormone released from the hypothalamus directly into the systemic circulation (bypassing the blood-brain barrier) as a direct response to stress caused by critical illnesses including ischaemic stroke [23].

Furthermore, copeptin acts on V1a, V1b, and V2 receptors after its release into the systemic circulation. V1b receptors are found in different parts of the brain including the cerebral cortex, subcortical structures like the hypothalamus, thalamus, and adenohypophysis. The effect of its stimulation at the level of the adenohypophysis causes adrenocorticotropic hormone (ACTH) to be released, which in turn causes cortisol, a stress hormone, to be released from the adrenal cortex [16]. In contrast to the finding in this study, Wendt and colleagues [28] found no statistically significant difference between serum copeptin levels among stroke patients and controls in their study. This may be because of the copeptin assay method used in this study.

The mean score of NIHSS in this study is 16 ± 10.7. The majority of the stroke patients had moderate stroke (40%) followed by severe stroke (31.3%). This study did not find any statistically significant difference between serum copeptin levels among the stroke severity categories (spearman rho p=0.076). It also did not demonstrate rising copeptin levels with increasing stroke severity. Likewise, the ability of serum copeptin to predict stroke severity with respect to NIHSS was very low in this study, with an AUC of 0.51 (95% CI: 0.36-0.65: p= 0.982). This is similar to a finding by Von Recum and colleagues, who reported a median NIHSS score of 7 (IQR- 3-11) in their ischaemic stroke cases and a median serum copeptin level of 19.1 pmol/L (IQR- 11.2-48.5) which was not statistically significant [29]. This is, however, in contrast to other studies that reported a statistically significant association between rising serum copeptin levels with stroke severity using NIHSS. This may be explained by their assay of serum copeptin shortly after symptom onset, made possible by likely early presentation of patients to the hospital as they reported that measurement of serum copeptin within 24 hours of stroke onset correlated significantly with stroke severity in their studies and was an independent prognostic indicator of outcome after three months of follow up [16]. A study by Spagnolello et al., whose stroke cases were recruited within 12 hours of symptoms onset, revealed persistently higher copeptin levels in their stroke patients at 24 Hours and similarly correlated to stroke severity [30]. The possible processes by which serum copeptin is thought to increase in severe acute ischaemic stroke include the presence of cerebral oedema that is contributed to by AVP release [31]. Other explanations include hyponatraemia, either from the syndrome of inappropriate antidiuretic hormone secretion (SIADH) or cerebral salt wasting syndrome, and fluid overload, both of which also stimulate AVP release [32].

Likewise, this study demonstrated higher levels of serum copeptin in larger infarct volumes and recorded a positive correlation, albeit weak, between infarct size measured on neuroimaging on admission with serum copeptin. Other studies have also demonstrated a similar rise, however, at a statistically significant level. A systematic review and meta-analysis conducted by Blek and colleagues to assess the diagnostic and prognostic value of copeptin in patients with acute ischaemic stroke, found in most studies reviewed that the initial infarct volume in the brain as determined by a CT or MRI scan and copeptin have a positive correlation with a rise in the level of this biomarker. This can be explained by the association between the development of cerebral oedema (with subsequent release of AVP) following focal restriction of blood flow as seen in acute ischaemic stroke. This suggests that a large infarct may likely result in a greater consequent cerebral oedema and hence, a higher AVP release. AVP release stimulates V1a and V2 receptors, which then cause platelet aggregation, vasoconstriction, and water retention with low plasma osmolality and hypovolemic or euvolemic hyponatraemia as consequences with resultant infarct volume expansion. This is the first study we are aware of that looked into the relationship between copeptin level, ischaemic stroke severity, and infarct volume in the country [22,33,34].

The limitations of the study include the relatively small sample size and the single-center design, which may potentially limit the generalization of the results. Secondly, the predictive value of copeptin in hospitalized stroke patients may be hampered by the connection between copeptin release and the body's stress responses as well as the possible rise in plasma copeptin levels in the presence of medical comorbidities.

## Conclusions

In this study, acute ischaemic stroke patients were found to have significantly elevated levels of serum copeptin compared to participants in the control group and a non-statistically significant rise with infarct size. Although, this did not have a prognostic value in predicting stroke severity (with NIHSS). This study, however, agrees that copeptin, an easily accessible molecule, appears to have an interesting potential as a biomarker in stroke, as evidenced by its rise in the acute phase of stroke and variations of findings from other studies. The finding from this study is intended to stimulate more research, potentially to explore the relationship between copeptin and acute stroke severity in larger studies as well as with other predictors of stroke outcome.
